# American Public Health Association

**Published:** 1896-08

**Authors:** 


### BUFFALO MEDICAL JOURNAL.

A Monthly Review of Medicine and Surgery.

*EDITORS:*

THOMAS LOTHROP, M. D. - - WM. WARREN POTTER, M. D.

All communications, whether of a literary or business nature, should be addressed to the managing editor: 284 FRANKLIN STREET, BUFFALO, N. Y.

VOL. XXXVI. AUGUST, 1896. NO. 1.

### AMERICAN PUBLIC HEALTH ASSOCIATION.

IT IS now becoming pretty generally known through announcements in the medical journals that the American Public Health Association will hold its twenty-fourth annual meeting at Buffalo, Tuesday, Wednesday, Thursday and Friday, September 15, 16, 17 and 18, 1896. This event will be of sufficient importance to justify somewhat extended comment at our hands at this time.

This association was organised, in 1872, by a few eminent sanitarians, who foresaw the importance of such a society that would bring within the fold of its membership the ablest men interested in public health and the prevention of disease. The founders included the names of Drs. Stephen Smith, Elisha Harris, Francis Bacon, Christopher C. Cox and John H. Rauch.

The marvelous growth of the association from a score or more of public-spirited men in 1872, to a membership of several thousand in 1896, whose residences are scattered over every nook and cranny of the United States, the Dominion of Canada and the Republic of Mexico, is indicative of its importance and the confidence it has inspired among the people. It has held meetings in every prominent city in these three several countries, and it has discussed almost every question pertaining to public health and preventive medicine. Nor is its membership limited, as many suppose, to the profession of medicine. In its list of membership may be found the names of lawyers, ministers, civil and sanitary engineers, health officers, teachers, plumbers, merchants and physicians; indeed, almost every profession, as well as many of the industries, is represented in its work. The only qualifications required for membership are good professional or business standing, an interest in hygiene and public sanitation, and the indorsement of two members of the association. The membership fee is $5.00 a year.

The publications of the association constitute in themselves a library upon sanitation, and are alone worth more than the cost of membership. Last year the association commenced to publish its transactions in the form of a quarterly journal, that is as handsome a specimen of the printing art as we have seen in many a day, and which may be bound at the end of each year into a book uniform with the preceding volumes. So much for the association itself.

Now let us speak of the Buffalo meeting. Of late it has become quite common for organisations, civic, professional and military, to appoint their annual conventions in Buffalo. The natural advantages of this city growing out of its geographical location, its great and ever-increasing railway facilities, its opportunities for pleasure by land and water, its growing commercial importance, its splendid hotel accommodations, its beautiful streets paved with asphaltum, lined by tall shade trees and skirted with beautiful residences, all conspire to make it one of the most attractive convention cities in the United States. Moreover, to all of this should be added the business enterprise and liberality of its large-hearted and public-spirited citizens, who are equally famous for their social attractiveness, which latter is chiefly attributable to the beauty and refinement of its women.

Many opportunities have been offered of late to test the liberality of our people, but, in our view, nothing has yet been presented with more direct claims upon their hearts and purses than the entertainment of the American Public Health Association during its approaching meeting. It is true we have entertained the educators and the press league and are about to receive the scientists; but neither education, nor science, nor newspaper can prove attractive to any human being in the absence of health. Of what avail is learning without the necessary health to apply it? The highest function of the modern physician is exercised in the prevention of disease. The state has recognised this in the establishment of boards of health all over the land, in the creation of medical examing boards in almost every commonwealth, and in the enactment of other laws that have for their object the protection and preservation of the health of its citizens.

Here now comes the American Public Health Association, whose object is to aid the state in carrying out the above-named purposes by studying and evolving the best method to gain the ends sought, and to economise the public outlay in developing the highest order of state and municipal sanitation. It is true it costs money to entertain this association, but the expenditure is an investment that yields the largest return of any known to us. We feel sure that every intelligent man and woman in Buffalo will recognise the truth of this statement, and that every one appealed to by the chairman of the local finance committee, Dr. Henry R. Hopkins, will respond with a liberality consistent with the cause and his own ability.

In another column we publish an excerpt from the Buffalo *Evening News*, showing the sanitary status of Buffalo, that we commend to the careful reading of everyone whose eye falls upon it. It speaks volumes for the management of the health department of our city, and is a truthful indorsement of the ability with which Dr. Ernest Wende has administered its affairs. Dr. Wende is chairman of the local committee of arrangements of the American Public Health Association, and as such is the officer chiefly responsible for its proper entertainment in this city next September. The executive committee, consisting of the chairmen of the several subcommittees and others, holds its meetings every Friday afternoon, from 4 to 5 o'clock, at the Ellicott Club. It is important that each member should attend this meeting with regularity.

It is proposed to entertain the association at Ellicott Square during its business sessions and where exhibit rooms will be provided by the local committee for the exhibition of hygienic and sanitary appliances. This exhibition will be under the direction of Dr. B. H. Daggett, chairman, to whom all applications for space and other information on the subject should be made.

In conclusion, we repeat that the importance of this meeting cannot be overestimated. This remark applies not to physicians alone but to business men in a larger sense. They are the many, physicians are few. The latter are quite willing to do the work and pay their money. We only ask the former to give us each in accordance with his own ability, remembering that he is paying to advance public health interests and reduce private doctors' bills. We feel assured that this will be the most important meeting ever held by this association and that it will be entertained by the citizens of Buffalo in a manner commensurate with hospitable dignity.

